# Antimicrobial Use and Manure Management Practices Among Commercial Chicken Farmers in Selected Regions of Tanzania: Gaps and Strategies for Mitigating Antimicrobial Resistance

**DOI:** 10.3390/ijerph23020226

**Published:** 2026-02-10

**Authors:** Fares J. Biginagwa, Alexanda Mzula, Erica Westwood, Sunday O. Ochai, Hezron E. Nonga, Anders Dalsgaard, Robinson H. Mdegela

**Affiliations:** 1 Department of Veterinary Medicine and Public Health, College of Veterinary Medicine and Biomedical Sciences, Sokoine University of Agriculture, Morogoro P.O. Box 3015, Tanzania; 2 Department of Microbiology, Parasitology & Biotechnology, College of Veterinary Medicine and Biomedical Sciences, Sokoine University of Agriculture, Morogoro P.O. Box 3015, Tanzania; 3 ICARS, International Centre for Antimicrobial Resistance Solutions, Ørestads Boulevard 5, 2300 Copenhagen, Denmark; 4 Antimicrobial Research Unit, College of Health Sciences, University of KwaZulu-Natal, Durban 4000, South Africa; 5 Department of Veterinary Tropical Diseases, Faculty of Veterinary Science, University of Pretoria, Pretoria 0110, South Africa; 6 Department of Veterinary and Animal Sciences, Faculty of Health and Medical Sciences, University of Copenhagen, Stigbøjlen 4, Frederiksberg C, 1870 Copenhagen, Denmark

**Keywords:** antimicrobial resistance drivers, manure management, commercial chicken

## Abstract

**Highlights:**

**Public health relevance—How does this work relate to a public health issue?**

- Antimicrobial resistance (AMR) is a major global public health threat that compromises the treatment of infectious diseases.
- Environmental exposure to antimicrobial residues and resistant bacteria through agricultural use of poultry manure represents an under-recognized pathway for AMR transmission.

**Public health significance—Why is this work of significance to public health?**

- Awareness may help to improve practices aiming to mitigate the spread of antimicrobial resistance, lowering the risk of hard-to-treat infections to humans and animals.
- This paper provides recommendations for strategic interventions focusing on improving manure handling and antimicrobial use practices to reduce environmental contamination, thereby safeguarding community health.

**Public health implications—What are the key implications or messages for practitioners, policymakers and/or researchers in public health?**

- Promoting validated manure treatment methods such as composting could substantially reduce environmental AMR risks while supporting safe organic fertilizer use.
- The findings highlight the need to integrate manure management into national AMR-frameworks and to enforce regulations requiring treatment of poultry manure before agricultural use.

**Abstract:**

The intensification of commercial chicken production has increased antimicrobial use and manure generation, raising concerns about residues and resistant pathogens entering the environment. Use of raw chicken manure can introduce antimicrobial compounds and resistance determinants into agricultural soils. This study examined antimicrobial use and manure management practices among chicken farmers in Morogoro, Dar es Salaam, and Unguja, and identified key gaps in national regulatory frameworks and their on-farm implementation. A structured questionnaire was administered to 351 farmers to assess the types and usage of antimicrobials and manure handling practices. Farmers reported using fourteen antibiotic classes and four antiparasitic agents, with tetracycline being the most frequently used (54.1%). Most farmers in Unguja (97.7%), Dar es Salaam (87.3%), and Morogoro (70.9%) either apply manure as fertilizer, sell it, or both. A large proportion (93.2%) reported that they do not process manure before use or sale, mainly due to lack of technical knowledge (77.4%). Awareness of the health hazards posed by pathogens (43.3%) and drug residues (57.5%) is low. This study revealed critical gaps, including weak regulatory enforcement, inadequate surveillance systems, limited cross-sectoral integration, irrational antimicrobial use, and limited farmer awareness. Strengthening regulatory frameworks, improving farmer training, and promoting safer manure management methods are recommended to reduce the environmental dissemination of antimicrobial residues and resistance.

## 1. Introduction

Antimicrobial resistance (AMR) is a global health concern with multifaceted origins, including the use, overuse, and misuse of antimicrobials (AMs) in livestock productions. With the increasing demand for food of animal origin, particularly eggs and meat, a wide variety of AMs are used to raise commercial chickens in many regions across the world [1,2]. Similarly, increased chicken production has led to a high generation of manure, which for years has been beneficially applied as organic fertilizer in crop farms and aquaculture ponds. However, the practice of using raw chicken manure for land fertilization is identified as one of the primary routes for the spread of AMs and resistant pathogens to the environment [3,4,5,6,7,8]. This is mainly because almost 90% of orally administered AMs are excreted in manure as original compounds or their metabolites [9,10,11]. Furthermore, AM residues in chicken manure that are spread on agricultural land may be mobile with the water flow in the soil and contaminate the environment, including surface and groundwater through leaching processes [12,13]. Studies have also revealed that AM residues from manure can persist in soils, be taken up by farmed crops and enter the food chain [14,15]. Moreover, AM contamination can promote the selection of resistant genes in microbial populations, leading to increased public health risks [16,17,18].

Manure from chickens is a valuable and cost-effective organic fertilizer, rich in essential micro- and macronutrients [13,19,20]. However, in many regions, particularly in low-income countries, manure is a largely produced but poorly regulated and often unmanaged byproduct [4,21,22]. For example, in 2024, it was estimated that Tanzania’s chicken population reached 56 million, which was predominately commercial layers and broilers [23]. It is estimated that a single chicken can produce 60 to 110 g of manure per chicken, depending on breed, age, diet, and weight [20,24]. This means that a population of 56 million could generate between 3640 and 6160 tons of manure per day. Given that commercial chicken farms are massive and mainly concentrated in urban and peri-urban areas with limited space for storage and dumping, managing such vast quantities of manure, which contain both chemical (such as AM residues) and biological (i.e., resistant bacteria) hazards, poses a significant challenge.

In Tanzania, most livestock keepers traditionally heap manure (meaning to pile) temporarily before disposal or land application [21]. This practice is unsafe because when such manure is used as fertilizer, it can lead to the spread of AM residues, pathogens, and other contaminants in the environment, risking human and animal health [6,8,25]. Despite increased attention to AMR in livestock, major gaps in research, regulation, and implementation persist within commercial chicken production systems. Existing studies have focused on AM usage patterns and the prevalence of resistant microbes in poultry and animal products [26,27], but there is lack of literature specifically linking AMU with manure management and their combined impact on environmental AMR. This study examined AMU and manure management practices among commercial chicken farmers in selected regions of Tanzania, emphasizing their potential role in the environmental dissemination of AMR. This study also reviewed existing policies and regulations to identify critical gaps and recommend intervention strategies designed to strengthen policy frameworks and promote better on-farm practices.

## 2. Materials and Methods

### 2.1. Study Areas

This study was carried out in the Dar es Salaam and Morogoro regions of mainland Tanzania, as well as Unguja in Zanzibar.

Dar es Salaam, the largest commercial city in Tanzania, is administratively divided into four municipalities (Kinondoni, Temeke, Ubungo, Kigamboni) and Ilala city council. It is the most populous city, with 5,383,728 inhabitants [28]. In Morogoro region, this study was conducted only in Morogoro municipality. Morogoro municipality has a population of 471,409 [28] and lies approximately 190 km to the west of Dar es Salaam. Notably, Dar es Salaam and Morogoro have a substantial number of large commercial poultry farms classed as sector 2 and sector 3 according to the Food and Agricultural Organization (FAO) [29]. Moreover, apart from the rising consumer demand for chicken products in Morogoro, significant poultry farming activities in the region are largely influenced by the highest demand for poultry products in the Dar es Salaam market. The third study region was Unguja, which is the largest and most populated city in Zanzibar, with a total population of 1,346,332 [28]. The island is approximately 105 km from Dar es Salaam. It is a notable area for commercial chicken farming, mainly influenced by the local population, traditional practices associated with dietary preferences and tourism-driven demands.

### 2.2. Study Design and Sample Size

The study areas were selected based on the presence of commercial chicken farms, particularly those with flocks of 200 or more broilers and layers, and their accessibility for field data collection. The purposive sampling approach was designed to capture insights from regions with substantial commercial poultry activity. A questionnaire-based cross-sectional study was conducted from July to October 2023. The questionnaires were administered to farm owners or any adult (18+ years) person responsible for farm management. Before administering the questionnaire, the purpose of the study was explained, and respondents were asked for their verbal informed consent to participate in this study.

The sample size was determined using the formula for finite populations according to Uakarn et al. [30]. Using a 5% margin of error and a 95% confidence level, we determined proportional sample sizes of 252, 55, and 44 farms for Dar es Salaam, Morogoro, and Unguja, respectively, based on the relative number of registered chicken farms in each region. The survey focused on farms raising broilers, layers, or both, with a minimum flock size of 200 chickens serving as the inclusion criterion.

### 2.3. Data Collection and Procedures

Data were collected using a structured questionnaire administered to farm owners or adult attendants. The questionnaire covered farm characteristics, respondents’ sociodemographic information, AMU (including frequency and reasons for use), and manure management practices. The questionnaire was translated into Swahili and pre-tested with three broiler and layer farmers who met the inclusion criteria to ensure clarity, validity, and relevance.

Before commencing data collection, all enumerators underwent a comprehensive training session to ensure consistency and accuracy in data gathering. The training covered the objectives of the study, ethical considerations, and detailed explanations of each section of the questionnaire. Enumerators were guided on how to approach respondents, obtain informed consent, and maintain confidentiality throughout the interview process. Practical exercises and mock interviews were conducted to familiarize the team with the questionnaire flow, clarify ambiguous questions, and standardize interview techniques. This preparation ensured that all data collectors had a uniform understanding of the survey tools and could effectively engage with farmers in both English and Swahili.

To help respondents in providing information that required advanced knowledge or to support recollection of AMU practices, several methods were applied. First, numerators displayed posters with pictures of locally available drugs during the interview to help respondents identify the drugs they had used within the past month. Second, respondents were asked to bring any leftover drugs for identification by enumerators, which were also recorded in case they were not already included on the posters. Third, enumerators asked respondents to recall any other drugs that were not listed from the first and second methods.

### 2.4. A Brief Review of Existing Policies and Regulations on Antimicrobial Use and Manure Management (2000–2025)

Thematic content analysis was conducted to systematically review national policies, laws, and regulatory documents related to AMU and manure management. The review period (2000–2025) was chosen to encompass the timeframe during which major health, veterinary, environmental, and agricultural regulatory frameworks relevant to AMU, AMR, and waste management were developed or substantially revised in Tanzania. Reviewed documents were obtained either physically from relevant government offices or accessed online via official government websites. Each document was carefully read and manually coded through iterative reading and comparison, with related codes grouped into themes to identify areas of alignment, overlap, and regulatory gaps across sectors. In total, 12 national policy and regulatory documents were reviewed.

The review of policy and regulatory frameworks governing AMU in poultry production focused on documents that regulate veterinary practice, prescription, and drug distribution. These included the Veterinary Act (2003) [31], Animal Diseases Act (2003) [32], and the Livestock Policy (2006) [33], which collectively defines professional responsibilities and disease control procedures. Additional emphasis was placed on the Tanzania Medicines and Medical Devices Act (2003, amended 2019) [34], and the National Action Plan on AMR (2017–2022 and 2023–2028) [35,36], which provide the legal framework for AM registration, quality assurance, prudent use, and AMR mitigation. These documents were reviewed to identify existing provisions and implementation gaps related to AM stewardship, prescription control, and post-market surveillance in the poultry sector. For manure management, the analysis concentrated on policies and environmental frameworks that regulate waste handling, composting, and fertilizer production. These included the Fertilizer Act, 2011 [37], and its Control Regulations, 2017 [38]; the National Environmental Policy, 2021 [39]; and the Environmental Management Act, 2004 [40], under which the Solid Waste Management Regulations, 2009, the Hazardous Waste Control Regulations, 2021, and relevant national guidelines for liquid waste management are implemented [40,41]. In addition, cross-sectoral frameworks, including the One Health Strategic Plan (2022–2027) [42], and the AMR Surveillance Framework (2018) [43], were examined to assess institutional coordination and the integration of manure management within One Health AMR control strategies.

### 2.5. Data Analysis

Data were cleaned, coded, and analyzed using IBM SPSS Statistics for Windows, Version 25.0 (IBM Corp., Armonk, NY, USA) and Microsoft Office Excel 2016. Descriptive statistics, including frequencies, percentages, medians, and means, were calculated to summarize sociodemographic characteristics, patterns of AM use, and other categorical variables such as production type (broilers, layers, or both), education level, and source of AM information. Pearson’s Chi-square test was used to assess associations between these categorical variables at a 5% significance level, after confirming that all underlying assumptions were met.

## 3. Results

### 3.1. Sociodemographic Characteristics of Respondents

As shown in Table 1, the majority of the participants were female (*n* = 253). The age of the participants ranged from 19 to 89 years, with an overall mean age of 49 ± 13 years. For analysis purposes, participants were categorized into three age groups: young adults (18–35 years old), middle-aged adults (36–59 years old), and elders (60 years old and above [28]). Most respondents were middle-aged adults (*n* = 214, 61%). In addition, the highest level of education for the majority of respondents was secondary (*n* = 156, 44.4%).

### 3.2. Demographic Characteristics of Commercial Chicken Farms

The demographic characteristics of the 351 surveyed commercial farms are summarized in Table 2. The production scale classifications used in this study are based on the framework adopted by the Food and Agriculture Organization of the United Nations for categorizing poultry production systems in Tanzania [44]. The size of layer farms ranged from 200 to 5000 chickens, with a median of 500 (IQR: 346–1400). Broiler farms ranged from 200 to 7000 chickens, with a median of 500 (IQR: 300–800). Broiler (*n* = 275, 78.3%) and layer (*n* = 25, 55.6%) farms were small-scale farms. In addition, a significant proportion of layer farmers (*n* = 33, 73.3%) and broiler farmers (*n* = 313, 96.6%) utilized the deep litter system in their production systems, as opposed to the battery cage system.

### 3.3. Health Status of Chickens on Farms

The reported chicken health status is summarized in Table 3. Participants were asked about the last time they encountered a sick chicken on their farms. The majority of respondents from broiler farms (72.5%), layer farms (57.1%) and farms raising both layers and broilers (76.5%) reported encountering a sick chicken within the past month of the visit. However, a few respondents from broiler farms (7.8%) and layers farms (3.6%) reported not having observed any clinical signs of disease in their flocks.

### 3.4. Antimicrobial Agents Used in Study Farms

A total of 14 antibiotics were used alone or in combination, and 4 types of antiparasitic agents were used in chicken farms (Table 4). The tetracycline class was found to be most used, either alone (*n* = 87, 24.8%) or in combination with another antibiotic class (*n* = 103, 29.3%). Amprolium (*n* = 43, 12.3%) was the most frequently used antiparasitic agent. It was also observed that 11.3% (*n* = 36) of chicken farms had neither used antibiotic nor antiparasitic agents in the past month.

### 3.5. Estimated Amount of Manure Production

Respondents were asked to provide estimates of daily manure production from their farms, regardless of how often they clean the chicken houses. Results for the estimated daily manure production in the study chicken farms are summarized in Table 5, revealing that most of the small- and medium-scale farms generate less than 20 kg (50.7%) and between 20 kg and less than 100 kg (34.8%) of manure per day, respectively.

### 3.6. Manure Management Practices Among Poultry Farmers

**Figure 1:**
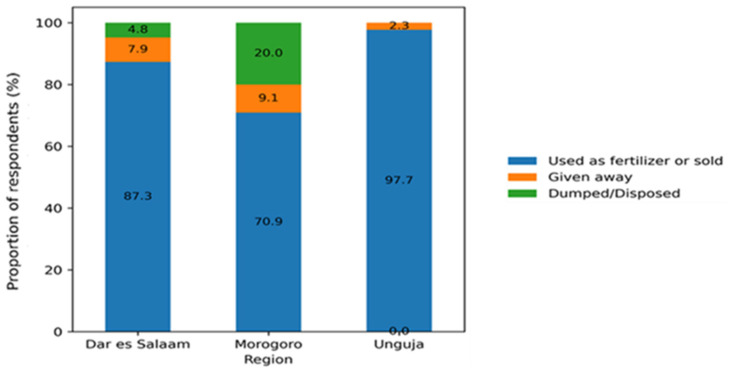
Manure management practices reported by commercial chicken farmers across study regions.

Farmers managed manure collected from their farms in several ways, as shown in Figure 1. Common practices included using it as fertilizer, selling it, or both. The results indicate that most farmers in Unguja (*n* = 43, 97.7%), Dar es Salaam (*n* = 220, 87.3%), and Morogoro (*n* = 39, 70.9%) utilize manure either as fertilizer, for sale, or both. Conversely, a smaller proportion of farmers reported disposing of manure by dumping it or giving it away to others in need.

### 3.7. Farmers’ Perceptions and Attitudes Toward Manure Management

**Figure 2:**
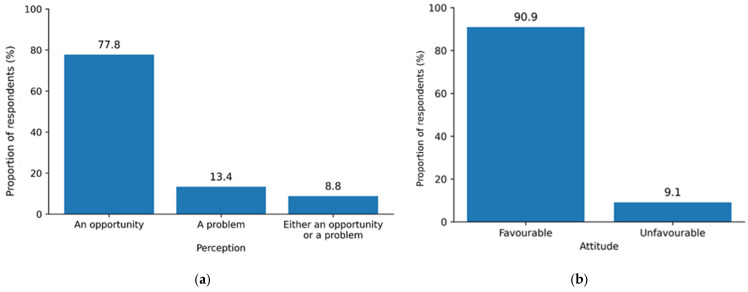
(**a**) Farmers’ perceptions of chicken manure and (**b**) farmers’ attitude towards manure management.

Mixed views were observed among farmers regarding manure generated on their farms. As shown in Figure 2a, many respondents (*n* = 273, 77.8%) appreciated that manure is an opportunity since they use it as fertilizer, sell it and give the manure to others. However, there were some farmers who regarded manure as a problem (*n* = 47, 13.4%), and others regarded it as both an opportunity or a problem (*n* = 31, 8.8%). Further analysis using a Chi-square test revealed a significant association (*p* = 0.001) between manure utilization practices and perceptions of manure. Similarly, respondents’ attitudes toward manure management were assessed using eight items covering specific aspects, including manure processing methods (such as composting and biogas production), availability of markets for both unprocessed and processed manure, and perceived public and environmental health challenges associated with manure handling. The items demonstrated acceptable internal consistency, with Cronbach’s alpha confirming adequate reliability (*α* = 0.762). Overall, the findings indicate that most farmers (90.9%) held a favorable attitude toward manure management (Figure 2b).

### 3.8. Manure Processing Practices Among Farmers

**Figure 3:**
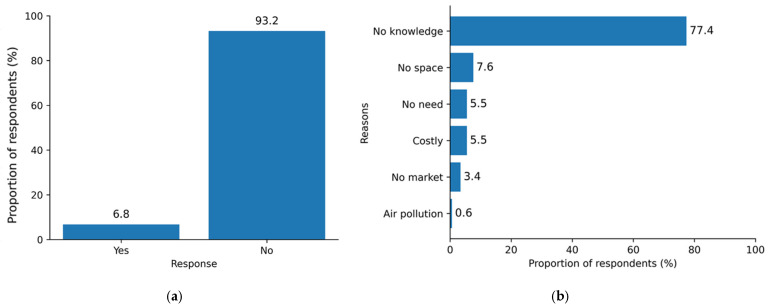
(**a**) Status of manure processing among farmers and (**b**) categorized reasons reported by farmers for not processing chicken manure.

Commercial chicken farmers were asked whether they processed the manure collected from their farms. The results (Figure 3a) showed that the majority of farmers (93.2%, *n* = 327) never engaged in manure processing. Additionally, the 327 respondents who did not process manure were asked to provide their reasons. Their responses were grouped into six categories based on similarities. The results (Figure 3b) indicate that 77.4% of respondents lacked knowledge about manure processing. The remaining participants cited various reasons, including insufficient space for processing, costs (time, equipment and facilities) and a perception that there was no need to process the manure, as they did not retain it for long after collection due to the availability of the market for unprocessed manure.

### 3.9. Awareness of Manure Processing Methods and Health Risks Associated with Manure Management

Respondents were asked to name any manure processing methods they were familiar with or had heard about. The results revealed that out of 351 respondents, 241 (68.7%) could not name a single method, while the remaining 110 (31.3%) mentioned various manure processing methods, including biogas production (*n* = 63), composting (*n* = 23) and both biogas and composting (*n* = 24). Despite a lack of knowledge about how manure is processed, there were few respondents (8.7%) who could name at least one processing method they had heard about. Moreover, the ability to name any existing manure processing methods was significantly associated with respondents’ sex (*p* = 0.002), indicating that males were generally more knowledgeable regarding manure management than females.

Three items were used to assess farmers’ awareness of the potential health implications related to manure management. As shown in Table 6, the Chi-square test indicated a lack of association between the items and the selected demographic characteristics, except for responses regarding the potential for manure to contain pathogens, which showed a significant correlation with education level (*p* = 0.034).

### 3.10. Key Policies and Regulation Gaps on Antimicrobial Use and Manure Management

Tanzania’s regulatory framework reveals several critical gaps in AMU and manure management. These identified gaps are summarized in Table 7.

## 4. Discussion

The use, overuse, and misuse of AMs, particularly in commercial chicken farming, are widely recognized as major contributors to AMR. Additionally, manure from treated chickens may retain a significant portion of unmetabolized AMs, serving as a potential reservoir for AMR spread in the environment when applied as soil fertilizer without proper treatment. This study surveyed 351 small- to large-scale commercial farms keeping broilers and layers in Dar es Salaam, Morogoro and Unguja, Tanzania. The aim was to assess AMU and the current manure management practices and their combined contribution to AMR in the environment. During the survey, most farmers reported encountering disease challenges within the past month of the visit. Disease incidence was relatively higher in broiler farms than in layer farms. To manage these disease challenges, over 90% of surveyed farms reported frequent use of AMs, mainly for treatment and prevention. Among the AM agents used by chicken farmers, the most common antibiotics were those in the tetracycline class, while amprolium was the most frequently used antiparasitic agent. The high frequency of tetracycline use among farmers in the current study is in line with other studies conducted in Tanzania [2,47], Uganda [48,49] and Ghana [50]. Moreover, another study in Tanzania reported that tetracycline accounts for over 60% of all veterinary AMs used due to its broad-spectrum efficacy, affordability, and widespread over-the-counter availability in agrovet outlets without strict prescription control [51,52]. The higher disease occurrence among broiler farmers compared to layer farmers can be attributed to differences in production systems and flock management practices. Broilers are raised under intensive conditions with high stocking densities, rapid growth rates, and short production cycles that increase stress and susceptibility to disease outbreaks [53,54].

In this study, most of the small- to large-scale farms reported that they could collect nearly 100 kg of chicken manure per day. Overall, estimated manure production showed a strong association with flock size. However, in very few farms, a mismatch between scale of production and the estimated amount of manure generated was observed. This could be because the amount of manure generated in chicken farms depends not only on chicken droppings but also on the amount and type of litter [24]. Considering that most surveyed farms employed a litter rearing system, the few small- and medium-scale farms that reported daily manure production of 100 kg and above could be due to variations in the amount and types of litter used. The respondents largely perceived chicken manure positively, citing economic benefits from its sale, use as fertilizer or giving it to others in need for various purposes. In contrast, a smaller percentage of farmers who perceived manure as a problem and could choose dumping instead showed concern about limited storage space, lack of reliable market and social conflict due to the incidences of odors and flies. The positive perception of manure among farmers significantly influences their behavior in utilizing it as a resource [55]. Therefore, the current study revealed that most respondents considered manure as a valuable and marketable byproduct of chicken farming, particularly for use as fertilizer.

This study found that most farmers do not process manure. Instead, the manure produced in most of the study farms is used as fertilizer, sold, or given away in its unprocessed form. Many respondents who never engaged in manure processing highlighted knowledge gaps about processing methods or their applications as a significant barrier. It was further revealed that most respondents were not familiar with manure processing methods as they could not mention any. Very few respondents were able to mention composting or biogas production as the common methods. This represents a concerning gap, as the application of raw manure may introduce AM residues and resistant microorganisms into agricultural soils [3,5,6,8]. These contaminants may subsequently be transported to surface- and groundwater through runoff and leaching or taken up by crops grown on amended soils [12,13]. Notably, even among the few respondents who processed manure, none did so with the aim of eliminating or reducing AMR determinants (AM residues, resistant microbes, or resistance genes). This finding aligns with a study from Dodoma that reported a high frequency of raw manure use for crop production [56]. Additionally, a study conducted in Bangladesh found that many farmers (69%) disposed of poultry farm litter on agricultural land without any processing [57]. Another similar finding in Cambodia showed that most chicken farmers did not process manure but temporarily stored it in heaps for use in home gardens or occasional sale [58]. This study also revealed that about half of the respondents were not aware of the potential health risks associated with manure management. Specifically, nearly half of respondents were not aware that manure could contain pathogens hazardous to human as well as animal health. In addition, over half were not aware that manure could contain AM residues. Similar knowledge gaps on the health risks of manure handling among livestock farmers were also reported in the previous studies conducted in Morogoro [21]. Specifically, a study conducted in Dodoma showed that most respondents were not aware that manure could contain drug residues that may subsequently accumulate in vegetables [56].

From a regulatory perspective, weak enforcement of existing regulations represents a critical barrier to effective AM stewardship in Tanzania. Therefore, there is an urgent need not only to revise policies and regulations governing AMU and manure management but also to substantially strengthen their implementation. Despite ongoing efforts to reduce AMU in commercial poultry farming, significant regulatory and enforcement gaps remain. Inadequate enforcement of the Veterinary Act, 2003, and its associated regulations has led to poor monitoring and control of AMU, manifested through infrequent inspections of veterinary drug outlets, weak adherence to record-keeping requirements, and widespread non-compliance with prescription-only provisions, as also reported in previous studies [47,59]. Moreover, there is a critical concern regarding the provision that indirectly allows farmers to administer treatments to their animals, regardless of their technical expertise. While this aims to empower farmers in managing livestock health, it may lead to misuse of AMs and exacerbate the spread of AMR.

While Tanzania’s agricultural policies promote organic farming and the use of manure as fertilizer [60], this study highlights a critical gap: raw manure from chickens treated with AMs is commonly applied without consideration of AMR risks. Despite the presence of regulatory bodies such as TFRA, existing frameworks do not explicitly regulate AM residues or resistance markers in organic fertilizers. Consequently, the current regulatory environment demonstrates substantial gaps in both manure processing technologies and certification standards for manure-derived products, particularly those intended for use as organic fertilizers. These shortcomings highlight the urgent need to revise the Fertilizer Act 2011 (Cap 378) along with its associated regulations, as well as the Standards Act 2009 (Cap 130) and its Tested Products Regulations. Such revisions would enhance manure processing to obtain safe organic fertilizer for sustainable agriculture. Additionally, the Environmental Management Regulations lack clarity on the issue of labeling for hazardous waste containing toxic substances. These regulations fail to explicitly classify manure as hazardous unless it is contaminated. Such ambiguity in the classification of hazardous waste exposes a regulatory gap that is not even adequately addressed by fertilizer regulations or hazardous waste regulations.

Our analysis underscores the necessity of reinforcing prescription-only access to AMs, establishing monitoring standards for organic fertilizers, and integrating manure management into AMR surveillance.

### 4.1. Recommendations

To address the gaps identified in this study, a set of multifaceted and evidence-based strategic interventions is required. Considering the high frequency of AMU and reports of unqualified drug administration, strengthening enforcement of the Veterinary Act and associated AMU guidelines should be prioritized through regular farm inspections, stricter licensing controls, and improved surveillance of veterinary AM distribution. Given that most farmers reported limited knowledge of prudent AMU and the risks associated with untreated manure, farmer-focused interventions, including targeted training on responsible AMU and safe manure handling, are essential to reduce misuse at the farm level. Additionally, the widespread application and sale of untreated manure observed in this study highlight the need to establish standardized monitoring systems for AMU and to integrate manure management into national AMR surveillance frameworks to enable early identification of environmental risks.

Based on the regulatory and implementation gaps identified, this study further recommends the preparation of policy briefs to inform the revision of existing regulations, particularly those requiring mandatory manure treatment prior to its use or sale as fertilizer, thereby supporting evidence-based policymaking. Finally, promoting validated manure processing technologies such as composting and biogas production, alongside the development of certification standards for safe organic fertilizers, can offer practical solutions for reducing the environmental dissemination of AMR and safeguard public, animal, and environmental health.

### 4.2. Limitations

This study has several limitations: First, AMU data were collected only to identify the common AMs used within the past month that are likely to disseminate into the environment through manure application; detailed information on dosing, frequency, treatment duration, and withdrawal compliance was not captured. Second, estimates of manure production were based on farmer reports and were not validated against measured or weighed values, which may introduce inaccuracies in estimating manure load. Third, the cross-sectional design limits causal inference, and convenience sampling based on accessibility may bias results toward better-managed farms, potentially underestimating poor practices. Fourth, the use of official registration lists as the sampling frame led to the exclusion of unregistered chicken farms. This could limit the representativeness and generalizability of the findings. Future research should employ longitudinal designs to track changes over time, validate manure production through direct measurements, and use more representative sampling frames to strengthen the generalizability and causal understanding of AMR risks associated with chicken manure.

## 5. Conclusions

Extensive use of AMs in commercial chicken farming, along with the production and application of raw manure potentially contaminated with residual AMs and resistant microbes, may pose serious threats to public health and environmental safety. This study underscores the urgent need for targeted educational initiatives to raise farmers’ awareness of the risks associated with raw manure use and AM misuse. Furthermore, regulatory frameworks must be reinforced to curb unqualified drug administration. Regarding manure management, existing policies and regulations should promote the adoption of safer manure management methods, such as composting and biogas production. Implementing these measures will not only mitigate the spread of AMR but also enhance soil fertility, resource efficiency, and environmental sustainability. Collectively, these measures provide a foundation for coordinated AMR mitigation and the promotion of safer, more resilient poultry production and sustainable agricultural systems in Tanzania.

## Figures and Tables

**Table 1:** Sociodemographic characteristics of respondents (*N* = 351).

Variable	Category	*n* (%)
Sex of respondent	Male	98 (27.9)
	Female	253 (72.1)
Age group (yrs.) (Mean: 49 ± 13)	Young adults (18–35)	58 (16.5)
	Middle-aged adults (36–59)	214 (61.0)
	Elders (≥60 years)	79 (22.5)
Education level	Informal	3 (0.9)
	Primary	109 (31.1)
	Secondary	156 (44.4)
	Tertiary	83 (23.6)
Experience (yrs.)–(IQR:6–12)	1–5 years	151 (43.0)
	6–10 years	96 (27.4)
	≥11 years	104 (29.6)

**Table 2:** Demographic characteristics of surveyed commercial farms.

Variable	Categories	*n* (%)
Type of production	Layer	28 (8.0)
	Broiler	306 (87.2)
	Both layer and broiler	17 (4.8)
Scale of production: Broilers	Small scale (≤1000 birds)	275 (78.3)
	Medium scale (1001–2000 birds)	36 (10.3)
	Large scale (>2000 birds)	12 (3.4)
Scale of production: Layers	Small scale (≤500 birds)	25 (55.6)
	Medium scale (501–2000 birds)	14 (31.1)
	Large scale (>2000 birds)	6 (13.3)
Production system: Broiler (*n* = 323)	Battery cage	7 (2.2)
	Deep litter	313 (96.6)
	Deep litter Battery cage	1 (0.3)
	Slated wire	2 (0.6)
Production system: Layer (*n* = 45)	Battery cage	7 (15.6)
	Deep litter	33 (73.3)
	Deep litter and Battery cage	5 (11.1)

**Table 3:** Last reported disease encounter in the study farms.

Production Type	Last Disease Occurrence	Frequency (%)
Broilers (*n* = 306)	<1 month	222 (72.5%)
	1–<7 months	60 (19.6%)
	Never been sick	24 (7.8%)
Layers (*n* = 28)	<1 month	16 (57.1%)
	1–<7 months	10 (35.7%)
	12 months and above	1 (3.6%)
	Never been sick	1 (3.6%)
Both layer and broiler (*n* = 17)	<1 month	13 (76.5%)
	1–<7 months	4 (23.5%)

**Table 4:** Antimicrobial agents used by respondents in the study farms.

Antimicrobials	Production Type			Total (*N* = 351)
Antibiotic class	Broiler	Layer	Broiler + Layer	*n* (%)
Tetracyclines	71	10	6	87 (24.8%)
Tetracycline (in combination with other AMs)	98	3	2	103 (29.3%)
Fluoroquinolones	44	2	0	46 (13.1%)
Sulphonamides	16	2	2	20 (5.7%)
Macrolides	2	2	0	4 (1.1%)
Aminoglycosides	2	0	1	3 (0.9%)
Penicilins	3	0	0	3 (0.9%)
Penicilin-colistin	4	0	0	4 (1.1%)
Antiparasites				
Amprolium	33	5	5	43 (12.3%)
Triazines (Toltrazuril)	2	0	0	2 (0.6%)
None	31	2	0	36 (11.3%)

**Table 5:** Estimates of daily manure production per day.

Estimated Amount of Manure	Production Type	Scale of Production			*n* (%)
		Small Scale	Medium Scale	Large Scale	
Less than 20 kg	Broiler	100	64	0	164
	Layer	6	1	0	7
	Broiler + layer	3	4	0	7
	Total				178 (50.7)
20 kg–<100 kg	Broiler	61	32	7	100
	Layer	9	6	1	16
	Broiler + layer	1	4	1	6
	Total				122 (34.8)
100 kg–<500 kg	Broiler	14	20	1	35
	Layer	2	1	1	4
	Broiler + layer	1	1	2	4
	Total				43 (12.3)
Over 500 kg	Broiler	0	3	4	7
	Layer	0	0	1	1
	Total				8 (2.3)

**Table 6:** Farmers’ awareness of the potential health risks associated with manure management.

Variables	Awareness Status		Sex	Age	Education Level	Experience
	Aware	Not Aware	X^2^ (*p*-Value)	X^2^ (*p*-Value)	X^2^ (*p*-Value)	X^2^ (*p*-Value)
**1.** Are there health concerns with manure?	181 (51.6)	170 (48.4)	0.680 (0.409)	0.267 (0.875)	5.783 (0.123)	1.104 (0.576)
**2.** Could manure contain pathogens?	199 (56.7)	152 (43.3)	1.136 (0.286)	0.132 (0.936)	8.661 (0.034) *	0.260 (0.878)
**3.** Could manure contain AM residues?	149 (42.5)	202 (57.5)	0.392 (0.531)	0.479 (0.787)	3.566 (0.312)	0.004 (0.998)

* Statistically significant association between variables.

**Table 7:** Key limitations in existing regulatory framework on AMU and manure management in Tanzania.

Analyzed Content and Policy/Regulatory Support	Identified Gaps
AMU Veterinary Act (2003) [31] The Code of Professional Conduct (2005) [45] Tanzania Medicines and Medical Devices Act (Cap 219, R.E. 2021) [34]	1. Lacks mechanisms to regulate informal providers 2. Farmer autonomy when deciding to administer treatments to their animals 3. Lack of training requirements on AMR mitigation strategies 4. Lack specific guidelines for prudent use, monitoring of AMR, or restrictions on non-therapeutic use in livestock.
Manure management Fertilizer Regulations (2011) [37] Standards (Tested Products) Regulations (2009) [46] Environmental Management Regulations (Hazardous Waste Control and Management) (2021) [40,41] National Environmental Policy (2021) [39]	1. No thresholds for biological contaminants (e.g., resistant pathogenic microbes) and chemical contaminants (e.g., residual AMs and heavy metals) in manure used as fertilizer or disposed in the environment. 2. Hazardous waste labelling requirements for toxic substances do not explicitly classify manure as hazardous unless it is contaminated. 3. In the Standards (Tested Products) Regulations (2009); testing procedures focus on nutrient content (N-P-K ratios) rather than microbial safety. No emphasis on manure processing to meet required standards. 4. Ambiguity in hazardous waste classification: Contaminated manure (e.g., containing veterinary drug residues), is not explicitly addressed by either fertilizer or hazardous waste regulations 5. Fragmented oversight: The Tanzania Fertilizer Regulatory Authority (TFRA) regulates fertilizers, and the National Environment Management Council (NEMC) oversees hazardous waste, but no interagency framework exists for manure management 6. Liability gaps: Hazardous waste generators are held accountable for environmental damage, whereas manure producers have no liability unless manure is classified as hazardous

## Data Availability

The data generated from this study might be shared with a valid request from the corresponding author.
